# The association of food insecurity and cardiometabolic risk factors was independent of body mass index in Iranian women

**DOI:** 10.1186/s41043-022-00322-w

**Published:** 2022-09-07

**Authors:** Maral Hashemzadeh, Maryam Teymouri, Mohammad Fararouei, Masoumeh Akhlaghi

**Affiliations:** 1 Department of Community Nutrition, School of Nutrition and Food Sciences, Shiraz University of Medical Sciences, Shiraz, Iran; 2 Department of Clinical Nutrition, School of Nutrition and Food Sciences, Shiraz University of Medical Sciences, Shiraz, Iran; 3 Department of Epidemiology, School of Health, Shiraz University of Medical Sciences, Shiraz, Iran

**Keywords:** Food insecurity, Cardiometabolic risk factors, Body mass index

## Abstract

**Background:**

Investigations on food insecurity have shown that food insecurity is inversely associated with health. We examined the association of food insecurity and cardiometabolic risk factors in women living in Shiraz, Iran.

**Methods:**

The cross-sectional study was performed on 190 females. Food insecurity was assessed by Household Food Insecurity Access Scale. Cardiometabolic risk factors including anthropometric characteristics, blood pressure, and serum glucose and lipids were measured. Metabolic syndrome score was calculated according to the criteria described for Iranian adults. The association of food insecurity and cardiometabolic risk factors was assessed by linear regression.

**Results:**

The prevalence of food security, and mild, moderate, and severe food insecurity was 42.6%, 40.5%, 15.8%, and 1.1%, respectively. Cardiometabolic risk factors worsened with increasing severity of food insecurity. Among the risk factors, body mass index (BMI) had the strongest association with food insecurity. After controlling demographic factors and BMI, fasting blood glucose, triglycerides, total, LDL, and HDL cholesterols, and metabolic syndrome score still showed significant associations with food insecurity (*P* < 0.01) but systolic and diastolic blood pressure were no longer associated with food insecurity after adjustment for BMI.

**Conclusion:**

Overall, although BMI was strongly associated with food insecurity, cardiometabolic risk factors including blood glucose, triglycerides, total, HDL, and LDL cholesterols, and metabolic syndrome score were associated with food insecurity independent of BMI, suggesting that other factors such as lifestyle and diet may have contributed to the exacerbated cardiometabolic risk in food insecure participants of this study. Future studies need to clarify underlying factors in the association of food insecurity and cardiometabolic risk factors.

## Introduction

Food insecurity is a public nutrition concern which affects every country in the world [1]. According to recent statistics, 10.5% of the US households in 2019 [2] and 20.8% of a representative sample from the UK in 2016 were food insecure [3]. Based on data of cross-sectional studies in Iran, 49.2% of Iranians are food insecure, 29.6% without and 19.2% with hunger [4].

Investigations have consistently shown that food insecurity is associated with poor health status [5]. Food insecure individuals have higher rates of hypertension, diabetes, coronary heart disease, stroke, depression, cancer, and asthma [6, 7] and are more likely to have a premature death [8]. A large retrospective cohort study showed that adults with marginal, moderate, and severe food insecurity died approximately 4.5, 6.2, and 9.4 years earlier than food-insecure individuals [8]. In this context, cardiovascular mortality has been found as an important cause of death in food insecure population, with a hazard ratio of 1.75 compared to the hazard ratio of 1.45 for all-cause mortality [9].

A number of investigations have indicated the association between cardiovascular risk and food insecurity (reviewed by Miguel et al.) [10]. However, only few studies have examined a wide range of cardiometabolic risk factors [11, 12], and these have produced conflicting results: while Hamedi-Shahraki and colleagues reported that the probability of having hypertriglyceridemia, general and abdominal obesity, and hypertension increased with exacerbation of food insecurity in Iranian women [11], Shariff et al. [12] found that food insecure Malaysian women were at lower risk for metabolic syndrome, abdominal obesity, elevated glucose, total and LDL cholesterol compared to food secure counterparts. It seems that the relationship between food insecurity and cardiometabolic factors differs between populations.

On the other hand, food insecurity and its relationship to health outcomes highly depends on sociocultural characteristics of the population, ecological environment, employment rates, resources, and economic and political condition of a community [13, 14]. Since these features differ from region to region, food insecurity investigations need to be conducted in different areas in order to determine health-related effects of food insecurity in different regions. Moreover, such investigations introduce directions for performing specific interventions to mitigate adverse consequences of food insecurity in each community. Hence, in the current study, we examined the relationship of food insecurity and cardiometabolic risk factors in a group of women living in Shiraz, Iran.

## Methodology

### Study design

This was a cross-sectional study conducted in spring and summer 2016 in Shiraz. A sample size of 190 was determined using a prevalence rate of 16.1% for moderate and severe food insecurity as reported by previous investigations [15], a confidence interval of 95%, and 5% margin of error. The sampling continued until the sample size was completed.

### Subjects

Participants were selected from attendees of primary health care centers located in all 9 municipal districts of Shiraz. Sampling was performed by multi-stage stratified cluster method from health care centers and through convenience sampling from attendees of each center. Inclusion criteria were as follows: females aged 20–55 years without medical conditions, such as hypertension, diabetes mellitus, renal or hepatic disorders, thyroid abnormalities, cancer, anorexia nervosa, pregnancy, and lactation. Also, they were not on special diets, such as caloric restriction diets. Exclusion criteria were cases without blood sampling. All the participants gave their written informed consent. The study was conducted in accordance with the Helsinki declaration and its later amendments. The project was approved by the ethics committee of Shiraz University of Medical Sciences (approval no. 1393–7271).

### Food insecurity

Food insecurity was assessed by Household Food Insecurity Access Scale (HFIAS) which is a tool for determining food insecurity in developing countries [16]. The questionnaire contains 9 items and has been translated and validated for use in Iranian communities [17]. The HFIAS scale scores the food insecurity from 0 to 27 and categorizes it into 4 levels of food secure (0–1), and mild (2–8), moderate (9–16), and severe (17–27) food insecure conditions.

### Anthropometric measurements

Weight was measured with minimal clothing using a digital scale (Glamor BS-801, Hitachi, China). Height was estimated without shoes with a tape fixed on a wall. Waist circumference was measured at the middle of the distance between the lowest rib and the iliac crest by using a non-stretchable tape. Body mass index (BMI) was calculated by dividing weight in kilograms by the square of height in meters.

### Blood pressure

Blood pressure was measured after 5 min rest with the use of a standing mercury sphygmomanometer (Alpk2, Japan). Participants were seated quietly for 5 min and blood pressure was measured twice with at least 5 min interval in between. The mean of two measurements was considered as the participant’s blood pressure.

### Biochemical measurements

Blood was taken after 12-h fasting. Serum was separated immediately and serum samples were stored in − 70 °C for later analysis. Glucose, triglycerides, total cholesterol, low-density lipoprotein (LDL) cholesterol, and high-density lipoprotein (HDL) cholesterol were quantified in serum samples by commercially available kits (Pars-Azmun, Tehran, Iran) and an auto-analyzer (BT 1500, Biotecnica Instruments, Italy). Metabolic syndrome score was computed according to the criteria described for Iranian adults by Iranian National Committee of Obesity [18]. According to this definition, metabolic syndrome is present if at least 3 of the following 5 criteria are met: waist circumference > 95 cm, systolic blood pressure > 130 mmHg or diastolic blood pressure > 85 mmHg, fasting glucose > 100 mg/dl, fasting triglycerides > 150 mg/dl, and high-density lipoprotein (HDL) cholesterol < 50 mg/dl for women.

### Statistical analysis

Data were analyzed by SPSS version 19 (SPSS Inc., Chicago, IL, USA). Normality of data was examined with Kolmogorov–Smirnov test and abnormally distributed data were log-transformed before being used in the analysis. Missing values were replaced using multiple imputation model based on the available data [19]. Missing-at-random assumption was used to generate 10 sets of imputed data, the pooled of which was used in the analysis.

For the analysis, due to low number of individuals in the severely food insecure level, participants in moderate and severe food insecure levels were combined in the moderate/severe food insecure level. Personal and household characteristics between food insecurity levels were compared with one-way analysis of variance (ANOVA) (for age) or chi-square (for categorical variables). Cardiometabolic risk factors were compared between levels of food insecurity by one-way ANOVA. Linear regression analysis was used to assess the relationship of food insecurity and cardiometabolic risk factors in unadjusted model and after controlling demographic characteristics (age, marital status, and educational level) in the model (model 1). BMI was added to the confounders to examine its contribution to the association of food insecurity and cardiometabolic risk factors (model 2). Results of regression analysis were expressed as standardized coefficient (*β*) to enable performing a comparison between cardiometabolic risk factors with different measurement scales. Statistical analysis was set at *P* < 0.05.

## Results

**Fig. 1:**
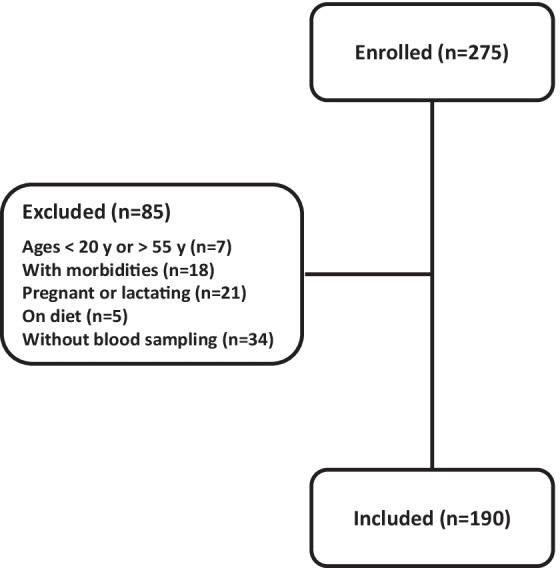
Flowchart of the study participants

The flow diagram of the study enrollment is presented in Fig. 1. A total of 275 females accepted to participate in the study but 85 were excluded after checking inclusion/exclusion criteria and thus 190 females aged 20–55 years were included. They had an average age of 35.2 ± 8.2 years, were mostly married (83.2%), housewife (72.6%), and educated in levels less than university (72.1%). According to the classification described in the Methods, 81 females (42.6%) were food secure, 77 (40.5%) were mild food insecure, 30 (15.8%) were moderately food insecure, and 2 (1.1%) were severely food insecure.

Characteristics of the participants in different food insecurity levels are presented in Table 1. Participants in different food insecurity levels did not differ in age and marital status but the educational level of the participants and participants’ household head was significantly higher in food secure group (*P* < 0.001). Distribution of cardiometabolic risk factors in different food insecurity levels is shown in Table 2. Weight, BMI, waist circumference, systolic and diastolic blood pressure, serum fasting glucose, triglycerides, total cholesterol, and LDL cholesterol increased while HDL cholesterol decreased across food insecurity levels (*P* < 0.001 to 0.009). The prevalence and intensity of overweight/obesity was lowest in food security and highest in moderate/severe food insecurity. The average of BMI in food secure participants was 25.2 kg/m^2^ which is almost normal and at the lowest overweight range but BMI of mild food insecure individuals was on average 28.1 kg/m^2^ which is in the middle of overweight range and that of moderate/severe food insecure participants was 33.1 kg/m^2^ which is in the range of obese class 1. Estimating the prevalence of obesity in different food insecurity levels revealed that 1.2%, 10.4%, and 28.1% of the participants in food security, mild insecurity, and moderate/severe insecurity groups had obesity class 2 and 0, 1.3%, 9.4% of them had obesity class 3, respectively.

**Table 1:** Participant’s characteristics based on food insecurity levels

	Secure (*n* = 81)	Mild insecure (*n* = 77)	Moderate/severe insecure (n = 32)	*P*^a^
Participant’s age (y)	34.9 ± 8.5	35.6 ± 8.5	35.2 ± 6.7	0.845
*Participant’s marital status*				
Single	12 (14.8)	11 (14.3)	9 (28.1)	0.173
Married	69 (85.2)	66 (85.7)	23 (71.9)	
*Participant’s education*				
School	38 (46.9)	68 (88.3)	32 (100)	< 0.001
Undergraduate	28 (34.6)	8 (10.4)	0 (0)	
Graduate	15 (18.5)	1 (1.3)	0 (0)	
*Household size, n (%)*				
1–2	17 (21.0)	14 (18.2)	5 (15.6)	0.33
3–4	54 (66.7)	52 (67.5)	18 (56.3)	
> 4	10 (12.3)	11 (14.3)	9 (28.1)	
*Household head education, n (%)*				
School	46 (56.8)	63 (81.8)	31 (96.9)	< 0.001
Undergraduate	27 (33.3)	13 (16.9)	1 (3.1)	
Graduate	8 (9.9)	1 (1.3)	0 (0)	
Household income, rials/mon	15,280,000	9,870,000	6,875,000	< 0.001

Scores of food insecurity are as follows: food secure (0–1), mild food-insecure (2–8), moderate/severe food-insecure (9–27)

Data are expressed as n (%) or means ± SD

^a^*P* value was determined by one-way analysis of variance for age and by chi-square for other variables

**Table 2:** Cardiometabolic risk factors in different food insecurity levels

	Secure (*n* = 81)	Mild insecure (*n* = 77)	Moderate/severe insecure (*n* = 32)	*P*^a^
Height (cm)	160.9 ± 6.3	159.4 ± 5.3	158.5 ± 4.9	0.077
Weight (kg)	65.3 ± 10.4	71.1 ± 12.1	83.1 ± 13.4	< 0.001
Body mass index (kg/m^2^)	25.2 ± 3.9	28.1 ± 5.1	33.1 ± 5.4	< 0.001
< 25	42 (51.9)	18 (23.4)	3 (9.4)	< 0.001
25 to < 30	30 (37.0)	36 (46.7)	4 (12.5)	
30 to < 35	8 (9.9)	14 (18.2)	13 (40.6)	
35 to < 40	1 (1.2)	8 (10.4)	9 (28.1)	
≥ 40	0 (0)	1 (1.3)	3 (9.4)	
Waist circumference (cm)	85.0 ± 10.4	87.5 ± 11.9	97.5 ± 12.0	< 0.001
Waist/hip ratio	0.86 ± 0.06	0.87 ± 0.06	0.90 ± 0.06	0.009
Systolic blood pressure (mmHg)	111.9 ± 11.6	116.4 ± 13.0	125.9 ± 14.0	< 0.001
Diastolic blood pressure (mmHg)	84.0 ± 9.5	88.5 ± 10.1	93.7 ± 11.3	< 0.001
Fasting blood glucose (mg/dL)	82.5 ± 12.0	87.7 ± 13.8	95.6 ± 21.8	0.001
Triglycerides (mg/dL)	133.2 ± 53.2	137.9 ± 48.9	179.3 ± 48.9	< 0.001
Total cholesterol (mg/dL)	174.3 ± 28.5	184.8 ± 39.6	230.6 ± 43.5	< 0.001
LDL cholesterol (mg/dL)	113.2 ± 22.8	120.4 ± 35.7	160.6 ± 37.9	< 0.001
HDL cholesterol (mg/dL)	47.2 ± 7.6	44.5 ± 8.1	39.2 ± 7.0	< 0.001
Metabolic syndrome score	1.85 ± 0.83	2.41 ± 1.20	3.48 ± 0.95	< 0.001

Scores of food insecurity are as follows: food secure (0–1), mild food-insecure (2–8), moderate/severe food-insecure (9–27)

Data are presented as means ± SD with the exception of BMI which has also been reported by number and percentage

^a^*P* values were determined by one-way analysis of variance (except for categorical BMI, for which chi-square was used)

Linear regression analysis demonstrated that all of the examined cardiometabolic risk factors associated with food insecurity (*P* < 0.01), with BMI having the strongest association (*β* = 0.555) (Table 3). The relationship remained statistically significant after controlling age, marital status, and educational level. The addition of BMI affected the results for diastolic blood pressure but results of other risk factors did not significantly change (*P* ≤ 0.01).

**Table 3:** Linear regression analysis assessing the association between levels of food insecurity and cardiometabolic risk factors

	Unadjusted model		Model 1^a^		Model 2^b^	
	*β*	*P*	*β*	*P*	*β*	*P*
Weight (kg)	0.513	< 0.001	0.480	< 0.001		
Body mass index (kg/m^2^)	0.555	< 0.001	0.512	< 0.001		
Waist circumference (cm)	0.384	< 0.001	0.369	< 0.001		
Waist/hip ratio	0.230	0.002	0.245	0.003		
Systolic blood pressure (mmHg)	0.389	< 0.001	0.407	< 0.001	0.223	0.010
Diastolic blood pressure (mmHg)	0.379	< 0.001	0.337	< 0.001	0.173	0.051
Fasting blood glucose (mg/dL)	0.313	< 0.001	0.300	0.001	0.322	0.001
Triglycerides (mg/dL)	0.305	< 0.001	0.271	0.003	0.284	0.006
Total cholesterol (mg/dL)	0.471	< 0.001	0.422	< 0.001	0.325	< 0.001
LDL cholesterol (mg/dL)	0.444	< 0.001	0.451	< 0.001	0.384	< 0.001
HDL cholesterol (mg/dL)	-0.369	< 0.001	-0.450	< 0.001	-0.408	< 0.001
Metabolic syndrome score	0.537	< 0.001	0.540	< 0.001	0.377	< 0.001

The associations were examined by linear regression. To compare the results of cardiometabolic risk factors with different measurement scales, the standardized coefficient (*β*) was reported

^a^Model 1 was adjusted for age, marital status, and educational level

^b^Model 2 was additionally adjusted for BMI

## Discussion

Results of this study showed that food insecurity was associated with exacerbated cardiometabolic risk factors. Among the risk factors, BMI had the strongest association with food insecurity. BMI seemed to be an important factor in the association of food insecurity with elevated systolic and diastolic blood pressure. However, the associations were independent of BMI for total, LDL and HDL cholesterol, fasting glucose, and metabolic syndrome score.

### Prevalence of food insecurity

The prevalence of food insecurity based on HFIAS tool in participants of this study was 57.4%, with 16.9% encountered with moderate to severe degrees of food insecurity. These results are comparable to the rates reported in previous investigations in Iran that were collected in a meta-analysis, in which the prevalence of food insecurity was 49% among households, 61% in women, and 65% in the elderly [20]. In rural areas, the insecurity rate was similar to that in households (40–50%) [21, 22] but in suburbs the rate was higher (82%) [23]

### Food insecurity and metabolic risk factors

Significant associations were found between food insecurity and the examined cardiometabolic risk factors and metabolic syndrome score. Food insecure participants had higher BMI and waist circumference, blood pressure, fasting glucose, triglycerides, and LDL cholesterol, and lower HDL cholesterol. These findings are in agreement with findings of recent systematic reviews [10] and meta-analyses [24] indicating a direct relationship of food insecurity (assessed by either food insecurity questionnaires or diet adequacy/diversity) with cardiometabolic risk factors, especially excess weight, hypertension, and dyslipidemias. These abnormalities in metabolic and cardiovascular risk markers gradually progress during the lifespan and eventually lead to chronic cardiovascular diseases [6], which results in increased rates of mortality from these diseases in food insecure groups [25, 26]. Morbidities that result from food insecurity impose medical costs to the households which in turn exacerbate the condition of food insecurity [27], establishing a vicious cycle between cardiometabolic diseases and food insecurity.

Despite the mostly common evidence for the positive association of food insecurity and cardiometabolic risk, a study in Malaysia showed an inverse association between food insecurity and a number of cardiometabolic risk factors including abdominal obesity [12]. The authors stated that the inverse association between food insecurity and cardiometabolic risk factors was due to persistent food insecurity in their participants which prevented overconsumption of energy-dense foods in periods of food affluence, as discussed in more details below.

### Food insecurity and risk of overweight/obesity

We found that weight and BMI increased along with worsening food insecurity. The prevalence and intensity of excess body weight was lowest in food secure participants and highest in those with moderate/severe insecurity. Among cardiometabolic risk factors, BMI had the highest association with food insecurity. In agreement, Holben and Taylor reported that among metabolic risk factors, overweight and central obesity had the strongest association with food insecurity in adolescents aged 12–18 years in the US [28]. The food insecurity-obesity paradox has been highlighted in previous investigations [29, 30]. In most cases of food insecurity, the amount of food consumption is not affected, but low-calorie nutritious foods are substituted with poor-quality, energy-dense foods that rectify hunger but promote overweight and obesity [1]. Moreover, insecure households may experience periods of hunger when food and money run out. At such times, to prevent intermittent hunger, families are pushed to choose relatively cheap high-calorie foods to enable to afford food for more prolonged periods of time [31]. Foods containing simple carbohydrates and oil are such foods, foods that lack adequate amounts of nutrients but are energy-dense and promote obesity. Furthermore, episodes of food deprivation may create a tendency in individuals to overeat to prolong satiation in periods of food affluence [31]. In this regard, studies have shown that both cases of mild and severe food insecurity may increase the likelihood of binge-eating disorder [32]. Although the reason of this binge-eating disorder is not clear, the involvement of food insecurity in its pathophysiology is likely. As stated above, binge eating may be used by food insecure individuals in periods of food abundance as a coping strategy to be prepared for future food deprivation. Anxiety and depression which occurs along with food insecurity [33] may also put individuals at risk of weight gain and obesity [34, 35]. Medication use (particularly anti-depressants) [34], physical inactivity [36], and emotional eating (an inclination to eat, especially energy-dense and palatable foods, in order to alleviate negative emotions) [37] are among mechanisms that have been suggested for the anxiety- or depression-induced overweight/obesity. Therefore, psychological treatments may be helpful for food insecure individuals in order to prevent overweight and obesity and the subsequent cardiometabolic diseases.

### Food insecurity-metabolic risk relationship independent of BMI

Obesity predisposes individuals to metabolic and cardiovascular diseases [38]. However, in the current study, the addition of BMI among the confounders did not affect the association between food insecurity and cardiometabolic risk factors. This suggests that factors other than excessive body weight have also contributed to the metabolic abnormalities of the food insecure participants of this study. Lifestyle, diet, exercise, and physical activity are factors affecting metabolic health. Epidemiologic surveys have shown that food secure individuals have healthier lifestyle, more physical activity, and less smoking habits than food insecure people [39, 40]. These factors are affected by health literacy which itself is affected by socioeconomic status. In higher socioeconomic classes, people have usually better job positions and higher income, but have also higher education and greater health and nutrition literacy, factors that can bring along healthier lifestyle and living habits. In this regard, a cross-sectional study on 2729 men and women aged 16–30 years in Canada indicated that those with lower health literacy had higher odds of food insecurity [41]. Another study on Australian population aged 18 years and older showed that insecure individuals exhibited less healthy dietary behaviors as a result of low food literacy. For instance, they were less likely to plan meals to include all food groups, think about healthy choices when deciding what to eat, use nutrition information panel to make food choices, cook meals at home using healthy ingredients, change recipes to make them healthier, and to drink sugar-sweetened beverages [42]. Food insecure people also had lower levels of education and employment status.

Whether lifestyle or diet-related factors have contributed to the association of food insecurity with cardiometabolic risk factors of this study needs to be determined in future investigations. In insecure groups, both participants and the household head had noticeably lower educational level than those in secure group: none of participants or household heads (except one) of moderate/severe insecure group had university education, and less than 20% of those in mild insecure group had university education, but about half of secure group had university education. Since individuals in higher educational levels usually possess better health literacy [43] it is likely that participants of food secure group had healthier diet and lifestyle habits, but this cannot be said with certainty based on these data. Future investigations will clarify this supposition.

### Strengths and limitations

There were strengths and limitations with this work. To the best of our knowledge, our study was among the few that have examined the relationship of food insecurity with a wide spectrum of cardiometabolic and metabolic syndrome components in healthy individuals. However, our participants were women, and so a comparison could not be performed between men and women. It is likely that due to female-specific personality or culturally imposed gender discrimination, women are influenced more severely than men by food insecurity aftermaths, such as poor diet quality and obesity [29, 44, 45]. On the other hand, compared to men, women spend more time at home and so are more likely to satisfy their hunger with high-energy low-nutritious foods, which results in their overweight [46]. Furthermore, due to fat accumulation during pregnancies [47] women are more prone to obesity than men. The contribution of health and nutrition literacy in lifestyle and dietary behaviors of participants was not clear. As an important confounder, health literacy affects lifestyle habits and food selection and thus may influence cardiometabolic risk. Future studies need to include evaluation of nutritional information and skills of food purchase and preparation in conditions of limited food budget. Smoking habits, alcohol consumption, physical activity, and psychologic conditions including depression and anxiety were not also assessed. These factors have potential to influence diet, eating behaviors, and cardiovascular risk. Future investigations may need to consider these points during study design and data collection.

## Conclusions

Overall, results of this study indicated a close relationship between food insecurity and cardiometabolic risk. Among the risk factors, BMI had the strongest association with food insecurity. The associations were mostly independent of demographic factors and BMI, suggesting that other factors such as lifestyle and diet may have contributed to the exacerbated cardiometabolic risk in food insecure individuals.

## Data Availability

Please contact author for data requests.

## Abbreviations

ANOVA: One-way analysis of variance
BMI: Body mass index
HDL: High-density lipoprotein
HFIAS: Household food insecurity access scale
LDL: Low-density lipoprotein
